# Effects of levels of clinical supervision during simulated ICU scenarios on resident learning and patient care: a qualitative study

**DOI:** 10.1186/cc11085

**Published:** 2012-03-20

**Authors:** D Piquette, M Mylopoulos, VR LeBlanc

**Affiliations:** 1 Sunnybrook Health Sciences Centre, Toronto, Canada; 2 SickKids Hospital, Toronto, Canada; 3 Wilson Centre, Toronto, Canada

## Introduction

Closer clinical supervision of residents is often perceived as a double-edged sword, improving patient safety but limiting resident participation in patient care. There has been little empirical research on the educational effects of closer supervision. We examined the impact of levels of clinical supervision on clinical learning and patient care during acute simulated resuscitation.

## Methods

Fifty-four ICU residents (PGY1 to 4) were randomly assigned to complete a simulated ICU scenario in one of three levels of supervision (physical proximity of supervising ICU fellow: distant, immediately available, direct). In-person and telephone interactions between participants were recorded and transcribed. We conducted an inductive thematic analysis of anonymized transcripts using constant comparison within and between scenarios. Distributed cognition theory was used as a framework to guide analysis.

## Results

Both distant and direct levels of supervision resulted in variable involvement of residents in patient care. A shift of control over patient care from residents to fellows often occurred regardless of the physical distance of the fellow. Direct supervision did not always result in decreased resident contributions. Fellows were found to facilitate more elaborated cognitive contributions from the residents during direct supervision. In addition, practicing in the presence of a supervisor was more likely to lead to timely feedback. However, a presence at the bedside allowed fellows to influence the nature of resident involvement by delegating specific tasks such as technical procedures. During distant supervision, fellows had to use residents as proxies to obtain information about patients and to deliver care, with potentially serious consequences: when residents' interpretations of the clinical information were problematic, the quality of fellows' clinical decisions was negatively affected. Higher cognitive work required of fellows during distant supervision appeared to limit their ability to invest cognitive resources in teaching.

## Conclusion

Level of clinical supervision was not the main determinant of resident engagement in patient care. Both distantly and directly supervised scenarios presented learning opportunities for residents. Given the observed negative effects of distant supervision on patient care, strategies to optimize unique learning opportunities offered by direct supervision should be investigated.

